# The association between polyunsaturated fatty acids and depression among Iranian postgraduate students in Malaysia

**DOI:** 10.1186/1476-511X-10-151

**Published:** 2011-08-24

**Authors:** Teymoor Yary, Sanaz Aazami

**Affiliations:** 1 Department of Nutrition, Faculty of Food Sciences and Engineering (FFSE), Science and Research Branch, Islamic Azad University (SRBIAU), Hesarak, Tehran, I.R.Iran

**Keywords:** Polyunsaturated fatty acids (PUFAs), depression, inflammation, neurotransmitter

## Abstract

**Background:**

The incidence of depression is expected to increase over the next 20 years, and many people will have to deal with it. It has been reported that up to 40% of university students experience levels of depression. Several negative consequences are associated with depression symptoms, such as memory impairment, suicide, and substance abuse. Recently, researchers have been studying possible associations between depression and polyunsaturated fatty acids (PUFAs), which may modify depression symptoms. The aim of the present study was to find an association between PUFA levels and depression among Iranian postgraduate students in Malaysia.

**Methods:**

This cross-sectional study was conducted in 2011 with 402 Iranian postgraduate students who were studying in Malaysia. The participants included 173 (43%) women and 229 (57%) men, and the mean age of the participants was 32.54 ± 6.22 years.

**Results:**

After adjustment for several potential confounders including sex, age, BMI, PUFAs, MUFAs, and SFAs, monthly expenses, close friends, living in campus, smoking, education, and marital status in a logistic regression model, an inverse relationship was found between depression symptoms and the dietary intake of PUFAs.

**Conclusion:**

We found an inverse association between PUFA intake and depression symptoms in Iranian postgraduate students in Malaysia. We, therefore, concluded that long-term intake of PUFAs may modify or prevent depression symptoms.

## Introduction

One of the most important health problems of the last century was depression [1]. The incidence of depression is expected to increase over the next 20 years, and many people will have to deal with it [2]. It has been reported that up to 40% of university students experience levels of depression [3]. Several negative consequences are associated with depression symptoms [4], such as memory impairment [5], suicide [6], and substance abuse [7]. Furthermore, it has been demonstrated that depression elevates the risks of several diseases such as coronary heart disease [8]; therefore, preventing and treating depression are necessary to protect students from its negative consequences.

Recently, researchers have been studying possible associations between depression and nutrient intake, which may modify depression symptoms. Based on the recommendations of several observational, epidemiological, and clinical studies, psychiatric disorders such as depression may be modified or amended by the intake of essential fatty acids [9]. These studies demonstrated that increased levels of polyunsaturated fatty acids (PUFAs) could enhance brain function and therefore stabilize depression symptoms [10].

Current treatments for depression cannot be targeted at the general population because depression is a silent disease that is difficult to diagnose. Thus, other treatments that may be applicable to the general population, such as nutritional assessments and interventions, are needed to prevent/modify depression. However, the results from previous studies are inclusive, and more investigations are needed. The aim of the present study was to find an association between PUFA levels and depression among Iranian postgraduate students in Malaysia.

## Materials and methods

### Study design and study subjects

This cross-sectional study in 2011 was conducted on a convenience sample of 425 Iranian postgraduate students, who were studying in Malaysia. The participants included 173 (43%) women and 229 (57%) men, and the mean age of the participants was 32.54 ± 6.22 years. Individuals with serious diseases such hyperthyroidism, hypertension, diabetes, and heart disease were excluded from the study, as such conditions may change lifestyle factors or modify the risk factors for depression. Students who had histories of mental illnesses or those taking antipsychotic drugs were excluded. Therefore, 23 students who did not meet the eligibility criteria were excluded from the study. This study was approved by the Scientific Counselor and Director of Iranian Students Affairs in South East Asia in Malaysia, and informed consent was obtained from all participants before enrollment.

### Determination of nutrient intake

A semi-quantitative food frequency questionnaire (FFQ) that was developed by Willet et al. [11], was used to measure typical and long-term food intake. The questionnaire has been validated and used to calculate dietary nutrient intake. Nutritionist IV software version 3.5.2 was used to analyze the levels of saturated fatty acids, monounsaturated fatty acids, and polyunsaturated fatty acids. The saturated and unsaturated fatty acids data were merged with sociodemographic and other variables for statistical analysis using SPSS version 18.

### Depression questionnaire

To measure depression symptoms, the self-administrated Center for Epidemiologic Studies (CES-D) questionnaire was employed [12]. The 20-item CES-D questionnaire measures the current level of depressive symptoms including appetite loss, sleep disorders, sadness, crying, feelings of loneliness, and fear. In the CES-D questionnaire, each question is answered using a 4-point Likert-type scale that ranges from 0 (rarely or none of the time (less than 1 day)) to 3 (most or all the time (5-7 days)), producing a total score of 0-60. The scoring is continuous, and a higher score indicates greater depressive symptoms. The cutoff score for depressive symptoms was 16.

### Statistical analysis

SPSS version 18 was used for the statistical analyses via parametric and nonparametric tests. Chi-square and ANOVA tests were run to analyze the data. A logistic regression analysis was performed to find an association between PUFA levels and depression symptoms, and the association was reported after adjustment for sex, age, body mass index (BMI), PUFAs, monounsaturated fatty acids (MUFAs), saturated fatty acids (SFAs), monthly expenses, close friends, living in campus, smoking, education, and marital status.

## Results

Table 1 shows the continuous and categorical variables of the participants based on the tertiles of PUFA intake. SFA and MUFA levels were significantly different among the tertiles of PUFA intake; SFA and MUFA levels were lower in tertile 1 than in tertiles 2 and 3. BMI values were not significantly different among the PUFA tertiles. Monthly expenses were significantly associated with PUFA intake; the mean monthly expenses were higher in the second and third tertiles than in the first tertile. Sex was not significantly different among the tertiles of PUFA intake. Several variables, including living in campus, years of studying, close friends, age, and smoking (current and former), were not associated with PUFA intake. Furthermore, there was no relationship between marital status and PUFA intake, although the first tertile featured fewer married subjects than did the other tertiles. Lastly, the results demonstrated that a high level of depression symptoms was associated with low levels of dietary PUFA intake; the prevalences of depression symptoms in the first, second, and third tertiles were 29.1%, 23.9%, and 38.1%, respectively.

**Table 1:** Characteristics of the subjects based on the tertile of PUFAs intake (N = 402)

Variables	T_1_	T_2_	T_3_	P
	(N = 134)	(N = 134)	(N = 134)	
Depression Symptoms, %	38.1	23.9	29.1	0.038
Living in Campus, %	41.0	47.0	38.8	0.372
Current Smoking, %	7.5	10.4	9.0	0.693
Former Smoking, %	4.5	4.5	3.0	0.771
Married, %	44.0	56.7	54.5	0.085
Close Friends	6.00 (4.50)	7.00 (5.00)	7.86 (5.5)	0.124
PUFAs (g/day)	7.18 ± 2.12	12.67 ± 1.73	27.84 ± 10.85	0.000
SFAs (g/day)	12.03 ± 6.20	17.92 ± 7.49	32.51 ± 17.72	0.000
MUFAs (g/day)	10.86 ± 4.09	17.43 ± 6.15	34.00 ± 19.95	0.000
Age (y)	32.32 ± 6.00	32.69 ± 6.44	32.60 ± 6.17	0.879
BMI (kg/m2)	24.06 ± 3.77	24.52 ± 4.72	24.40 ± 4.23	0.669
Sex, Female/Male	45.0/54.5	36.6/63.4	47.0/53.0	0.175
Studying (y)	20.12 ± 2.11	20.45 ± 2.35	20.53 ± 2.50	0.311
Monthly Expenses ($)	761.10 ± 500.60	922.04 ± 447.94	970.67 ± 489 ± 61	0.001

T = Tertile; y = Year; SFAs = Saturated fatty acids; MUFAs = Monounsaturated fatty acids; PUFAs = Polyunsaturated fatty acids; BMI = Body mass index.

Further statistical analyses in this study were conducted regarding the association between PUFA intake and depression symptoms. After adjustment for all other variables in a logistic regression model, including sex, age, BMI, PUFAs, MUFAs, SFAs, monthly expenses, close friends, living in campus, smoking (current and former), education, and marital status, there was an inverse association between depression symptoms and the second tertile of PUFA intake (Table 2).

**Table 2:** The association between PUFAs intake and depression

Variables	OR*	95% CI	p
PUFAs			
Tertile 1	1.0	-	-
Tertile 2	0.51	0.29-0.90	0.014
Tertile 3	0.75	0.38-1.48	0.407

*Adjusted for sex, age, BMI, PUFAs, MUFAs, SFAs, monthly expenses, close friends, living in campus, smoking, education, and marital status.

## Discussion

To the best of our knowledge, this was the first study to investigate an association between depression and PUFA intake among Iranian students in Malaysia. After adjustment for several potential confounders including sex, age, BMI, PUFAs, MUFAs, and SFAs, monthly expenses, close friends, living in campus, smoking, education, and marital status in a logistic regression model, an inverse relationship was found between depression symptoms and the dietary intake of PUFAs.

Our findings extend the results of several studies that investigated a relationship between PUFAs and depression [13-15]. A study conducted in a population above 15 years of age reported that the consumption of fish, which is rich in PUFAs, was protective against mental health disease [16]. Researchers in Australia also indicated that a diet rich in omega-3 fatty acids improved mood and cognitive performance [17]. A recent meta-analysis reported that lower levels of both eicosapentaenoic acid (EPA) and docosahexaenoic acid (DHA) were associated with depression [18].

Although the mechanism of the association between PUFA intake and depression is not completely clear, several neurophysiological mechanisms may explain how PUFAs control or modify depression symptoms. One of the mechanisms is that neurotransmission in patients with depression could improve with increased dietary intake or supplementation of PUFAs. It has been demonstrated that high PUFA intake, including DHA and EPA, was associated with increased levels of dopamine and dopamine D2 receptors in the frontal cortex of the rat brain [19]. In addition, Delion et al. reported that low intake of PUFAs increase the number of 5-hydroxytryptamine (5-HT_2_) receptors [20]. It has been reported 5-HT_2 _receptors may be involved in the pathophysiology of depression [18,21]. Researchers have emphasized a relationship between dietary alpha-linolenic acid (ALA), in particular, and the control of depression, as they believe this nutrient can restart dopaminergic neurotransmission in the brain [22]. In addition, increased dietary intake of ALA stimulates both cholinergic and serotonergic neurotransmission [23,24].

Omega-3 fatty acid insufficiency reduces phosphatidylserine (PS) levels in the rat brain cortex, brain mitochondria, and olfactory bulb by at least 30% [25]; PS has antidepressant properties [26,27], and supplementation of this nutrient can improve depression [19]. Additionally, insufficient dietary intake of omega-3 fatty acids was associated with a reduction in glucose uptake by brain cells and cytochrome oxidase activity [28]; both glucose uptake and cytochrome oxidase activity are indicators of neuronal functional activity. Moreover, deregulation of blood-brain barrier may occur due to PUFA deficiency, which may lead to a decrease in the transport of amino acids and glucose to the brain [29].

Another mechanism by which PUFAs may influence depression is through inflammatory modulation [30], which is induced via glial cells [31]. A cluster of cytokines including interleukin-1 beta (IL-1β), IL-2, IL-6, interferon-gamma, and tumor necrosis factor alpha (TNFα) have been reported to correlate with depression symptoms [32-34]. These cytokines have been found to negatively affect the central nervous systems via several pathways such as downregulation of the hypothalamic-pituitary axis, neurotransmitter metabolism, and neurotransmitter mRNA levels as well as decreased neurotransmitter precursor availability [33,35,36].

It has been demonstrated that omega-3 fatty acids may modify depression symptoms by inhibiting proinflammatory cytokines [37], particularly IL-1β and TNFα [38]. Although it is unclear how omega-3 fatty acids regulate these cytokines, omega-3 fatty acids may control some substances involved in inflammatory processes such as prostaglandin E2, thromboxane A2, and histamine [33,35]. Furthermore, omega-3 fatty acids influence the activities of the hypothalamic-pituitary-adrenal axis and corticosteroid hormones [33,35].

## Limitation of the study

This study was a cross-sectional study, and thus, this design did not permit us to assess causal relationships. For example, depression symptoms may lead to a loss of appetite and result in reduced consumption of foods and PUFAs, or studying in a foreign country (Malaysia) away from family may have caused depression in our study population. The last potential limitation is that plasma PUFA level was not measured by the present study and which may suggests avenues for future studies.

## Conclusion

We found an inverse association between PUFA intake and depression symptoms in Iranian postgraduate students in Malaysia. We, therefore, concluded that long-term intake of PUFAs may modify or prevent depression symptoms.

## Abbreviations

ALA: Alpha-linolenic acid; BMI: Body mass index; CES-D: Center for Epidemiologic Studies; DHA: Docosahexaenoic acid; EPA: Eicosapentaenoic acid; FFQ: Food frequency questionnaire; IL-1β: Interleukin-1 beta; MUFAs: Monounsaturated fatty acids; PUFAs: Polyunsaturated fatty acids; SFAs: Saturated fatty acids; TNFα: Tumor necrosis factor alpha

## References

[B1] Ball HA Siribaddana SH Kovas Y Glozier N McGuffin P Sumathipala A Hotopf M Epidemiology and symptomatology of depression in Sri Lanka: A cross-sectional population-based survey in Colombo District Journal of Affective Disorders 2010 123 188 196 10.1016/j.jad.2009.08.014 19762085 PMC2946561

[B2] Christina C George T Gerassimos S Theodora P Nikos G Vasiliki M George L Antigoni M Evaggelia G Charalambos M Fish Consumption Moderates Depressive Symptomatology in Elderly Men and Women from the IKARIA Study Cardiology Research and Practice 2010 10.4061/2011/219578 PMC3010635 21197433

[B3] Khawaja NG Bryden KJ The development and psychometric investigation of the university student depression inventory Journal of Affective Disorders 2006 96 21 29 10.1016/j.jad.2006.05.007 16784780

[B4] Demir T Karacetin G Demir DE Uysal O Epidemiology of depression in an urban population of Turkish children and adolescents Journal of Affective Disorders 2011 in press 10.1016/j.jad.2011.05.041 21683451

[B5] Gunther T Holtkamp K Jolles J Herpertz-Dahlmann B Konrad K Verbal memory and aspects of attentional control in children and adolescents with anxiety disorders or depressive disorders Journal of Affective Disorders 2004 82 265 269 10.1016/j.jad.2003.11.004 15488256

[B6] Nrugham L Larsson B Sund AM Predictors of suicidal acts across adolescence: Influences of familial, peer and individual factors Journal of Affective Disorders 2008 109 35 45 10.1016/j.jad.2007.11.001 18096243

[B7] Lubman DI Allen NB Rogers N Cementon E Bonomo Y The impact of co-occurring mood and anxiety disorders among substance-abusing youth Journal of Affective Disorders 2007 103 105 112 10.1016/j.jad.2007.01.011 17291589

[B8] Yary T Soleimannejad K Rahim A Kandiah M Aazami S Poor J Wee T Aazami G Contribution of diet and major depression to incidence of acute myocardial infarction(AMI) Lipids in Health and Disease 2010 9 133 10.1186/1476-511X-9-133 21087475 PMC2994859

[B9] Freeman MP Hibbeln JR Wisner KL Davis JM Mischoulon D Peet M Keck PE Jr Marangell LB Richardson AJ Lake J Omega-3 fatty acids: evidence basis for treatment and future research in psychiatry Journal of Clinical Psychiatry 2006 67 1954 1967 10.4088/JCP.v67n1217 17194275

[B10] Colangelo LA He K Whooley MA Daviglus ML Liu K Higher dietary intake of long-chain [omega]-3 polyunsaturated fatty acids is inversely associated with depressive symptoms in women Nutrition 2009 25 1011 1019 10.1016/j.nut.2008.12.008 19195841 PMC2798585

[B11] Willett WC Sampson L Stampfer MJ Rosner B Bain C Witschi J Hennekens CH Speizer FE Reproducibility and validity of a semiquantitative food frequency questionnaire American Journal of Epidemiology 1985 122 51 65 4014201 10.1093/oxfordjournals.aje.a114086

[B12] Radloff LS The CES-D scale: A self-report depression scale for research in the general population Applied Psychological Measurement 1977 1 385 10.1177/014662167700100306

[B13] Tanskanen A Hibbeln JR Tuomilehto J Uutela A Haukkala A Viinamaki H Lehtonen J Vartiainen E Fish consumption and depressive symptoms in the general population in Finland Psychiatric Services 2001 52 529 10.1176/appi.ps.52.4.529 11274502

[B14] Timonen M Horrobin D Jokelainen J Laitinen J Herva A RSsSnen P Fish consumption and depression: the Northern Finland 1966 birth cohort study Journal of Affective Disorders 2004 82 447 452 15555697 10.1016/j.jad.2004.02.002

[B15] Hakkarainen R Partonen T Haukka J Virtamo J Albanes D Lonnqvist J Is low dietary intake of omega-3 fatty acids associated with depression? American Journal of Psychiatry 2004 161 567 10.1176/appi.ajp.161.3.567 14992986

[B16] Silvers KM Scott KM Fish consumption and self-reported physical and mental health status Public Health Nutrition 2007 5 427 431 10.1079/phn2001308 12003654

[B17] Lombard CB What is the role of food in preventing depression and improving mood, performance and cognitive function? Medical Journal of Australia 2000 173 104 105 10.5694/j.1326-5377.2000.tb139438.x 11149371

[B18] Lin PY Huang SY Su KP A meta-analytic review of polyunsaturated fatty acid compositions in patients with depression Biological Psychiatry 2010 68 140 147 10.1016/j.biopsych.2010.03.018 20452573

[B19] Chalon S ion-Vancassel S Belzung C Guilloteau D Leguisquet AM Besnard JC Durand G Dietary fish oil affects monoaminergic neurotransmission and behavior in rats The Journal of Nutrition 1998 128 2512 9868201 10.1093/jn/128.12.2512

[B20] Delion S Chalon S Guilloteau D Besnard JC Durand G Linolenic Acid Dietary Deficiency Alters Age Related Changes of Dopaminergic and Serotoninergic Neurotransmission in the Rat Frontal Cortex Journal of Neurochemistry 1996 66 1582 1591 8627314 10.1046/j.1471-4159.1996.66041582.x

[B21] Laino CH Fonseca C Sterin-Speziale N Slobodianik N Reinqs A Potentiation of omega-3 fatty acid antidepressant-like effects with low non-antidepressant doses of fluoxetine and mirtazapine European Journal of Pharmacology 2010 648 117 126 10.1016/j.ejphar.2010.08.047 20826148

[B22] Kodas E Vancassel S Lejeune B Guilloteau D Chalon S Reversibility of n-3 fatty acid deficiency-induced changes in dopaminergic neurotransmission in rats Journal of Lipid Research 2002 43 1209 12177165

[B23] Aid S Vancassel S Poumes-Ballihaut C Chalon S Guesnet P Lavialle M Effect of a diet-induced n-3 PUFA depletion on cholinergic parameters in the rat hippocampus The Journal of Lipid Research 2003 44 1545 10.1194/jlr.M300079-JLR200 12754277

[B24] Kodas E Galineau L Bodard S Vancassel S Guilloteau D Besnard JC Chalon S Serotoninergic neurotransmission is affected by n-3 polyunsaturated fatty acids in the rat Journal of Neurochemistry 2004 89 695 702 10.1111/j.1471-4159.2004.02401.x 15086526

[B25] Hamilton J Greiner R Salem N Kim HY n- 3 Fatty acid deficiency decreases phosphatidylserine accumulation selectively in neuronal tissues Lipids 2000 35 863 869 10.1007/S11745-000-0595-x 10984109

[B26] Brambilla F Maggioni M Blood levels of cytokines in elderly patients with major depressive disorder Acta Psychiatrica Scandinavica 1998 97 309 313 10.1111/j.1600-0447.1998.tb10005.x 9570493

[B27] Maggioni M Picotti GB Bondiolotti GP Panerai A Cenacchi T Nobile P Brambilla F Effects of phosphatidylserine therapy in geriatric patients with depressive disorders Acta Psychiatrica Scandinavica 1990 81 265 270 10.1111/j.1600-0447.1990.tb06494.x 1693032

[B28] da Silva AX Lavialle F Gendrot G Guesnet P Alessandri JM Lavialle M Glucose transport and utilization are altered in the brain of rats deficient in n-3 polyunsaturated fatty acids Journal of Neurochemistry 2002 81 1328 1337 10.1046/j.1471-4159.2002.00932.x 12068080

[B29] Ziylan ZY Bernard GC Lefauconnier JMA Durand GA Bourre JME Effect of dietary n-3 fatty acid deficiency on blood-to-brain transfer of sucrose,[alpha]-aminoisobutyric acid and phenylalanine in the rat Neuroscience Letters 1992 137 9 13 10.1016/0304-3940(92)90286-G 1625821

[B30] Schmitz G Ecker J The opposing effects of n-3 and n-6 fatty acids Progress in Lipid Research 2008 47 147 155 10.1016/j.plipres.2007.12.004 18198131

[B31] Shapiro H Could n-3 polyunsaturated fatty acids reduce pathological pain by direct actions on the nervous system? Prostaglandins, Leukotrienes and Essential Fatty Acids 2003 68 219 224 10.1016/S0952-3278(02)00273-9 12591006

[B32] Mamalakis G Tornaritis M Kafatos A Depression and adipose essential polyunsaturated fatty acids Prostaglandins, Leukotrienes and Essential Fatty Acids 2002 67 311 318 10.1054/plef.2002.0435 12445491

[B33] Logan AC Neurobehavioral aspects of omega-3 fatty acids: possible mechanisms and therapeutic value in major depression Alternative Medicine Review 2003 8 410 425 14653768

[B34] Boissonneault GA Chow CK Dietary fat, immunity and inflammatory disease 2000 New York: Marcel Dekker Inc

[B35] Song C Li X Kang Z Kadotomi Y Omega-3 Fatty Acid Ethyl-Eicosapentaenoate Attenuates IL-1 -Induced Changes in Dopamine and Metabolites in the Shell of the Nucleus Accumbens: Involved with PLA2 Activity and Corticosterone Secretion Neuropsychopharmacology 2006 32 736 744 16794572 10.1038/sj.npp.1301117

[B36] Logan AC Omega-3 fatty acids and major depression: A primer for the mental health professional Lipids Health Dis 2004 3 25 10.1186/1476-511X-3-25 15535884 PMC533861

[B37] Maes M Smith RS Fatty acids, cytokines, and major depression Biological Psychiatry 1998 43 313 9513744 10.1016/s0006-3223(97)00401-0

[B38] Logan AC Neurobehavioral aspects of omega-3 fatty acids: possible mechanisms and therapeutic value in major depression Alternative Medicine Review 2003 8 410 425 14653768

